# Nitric oxide contributes to methane-induced osmotic stress tolerance in mung bean

**DOI:** 10.1186/s12870-018-1426-y

**Published:** 2018-09-24

**Authors:** Yihua Zhang, Jiuchang Su, Dan Cheng, Ren Wang, Yudong Mei, Huali Hu, Wenbiao Shen, Yaowen Zhang

**Affiliations:** 1 College of Life Sciences, Laboratory Center of Life Sciences, Nanjing Agricultural University, Nanjing, 210095 China; 2 Institute of Botany, Jiangsu Province and Chinese Academy of Sciences, Nanjing, 210014 China; 3 Institute of Agricultural Products Processing, Jiangsu Academy of Agricultural Sciences, Nanjing, 210014 China; 4 Crop Research Institute, Shanxi Academy of Agricultural Sciences, Taiyuan, 030031 China

**Keywords:** Methane, *Vigna radiate*, Osmotic stress, Nitric oxide, Redox homeostasis

## Abstract

**Background:**

Osmotic stress is a major abiotic stress limiting crop production by affecting plant growth and development. Although previous reports discovered that methane (CH_4_) has a beneficial effect on osmotic stress, the corresponding downstream signal(s) is still elusive.

**Results:**

Polyethylene glycol (PEG) treatment progressively stimulated the production of CH_4_ in germinating mung bean seeds. Exogenous CH_4_ and sodium nitroprusside (SNP) not only triggered nitric oxide (NO) production in PEG-stressed plants, but also alleviated the inhibition of seed germination. Meanwhile, amylase activity was activated, thus accelerating the formation of reducing sugar and total soluble sugar. Above responses could be impaired by NO scavenger(s), suggesting that CH_4_-induced stress tolerance was dependent on NO. Subsequent tests showed that CH_4_ could reestablish redox balance in a NO-dependent fashion. The addition of inhibitors of the nitrate reductase (NR) and NO synthase in mammalian (NOS), suggested that NR and NOS-like protein might be partially involved in CH_4_-alleviated seed germination inhibition. *In vitro* and scavenger tests showed that NO-mediated *S*-nitrosylation might be associated with above CH_4_ responses.

**Conclusions:**

Together, these results indicated an important role of endogenous NO in CH_4_-enhanced plant tolerance against osmotic stress, and NO-regulated redox homeostasis and *S*-nitrosylation might be involved in above CH_4_ action.

## Background

Mung bean (*Vigna radiata* L.) is consumed in large quantities in Asia due to its desirable taste and high nutrition value [1]. It is a good source of vitamins (A, B, C and E), minerals, and proteins with essential amino acids [2]. Mung bean has high medicinal function of curing diarrhea, headaches, edema, and eye problems [3]. However, the limited plant growth and crop production of mung bean widely exist because many regions of Asia are under osmotic stress.

Osmotic stress means that the water available potential is limited. Thus, sensing and signaling during water deficit stress might play key roles in plant water status, and bring about quick changes in gene expression [4]. Generally, polyethylene glycol (PEG)-6000 is considered as an applicable solute because of its properties of mimicking osmotic stress, which results in the inhibition of plant growth and development [5]. The water deficit caused by drought or osmotic stress could usually result in many changes in plant physiological processes [5, 6]. One of these changes is associated with the overproduction of reactive oxygen species (ROS) and thereafter oxidative damage, both of which have impacts on peroxidation of membrane lipids and the loss of plasma membrane integrity [7–9].

More importantly, perception and transduction of the stress-induced gaseous signaling molecules, including nitric oxide (NO), hydrogen sulfide (H_2_S), carbon monoxide (CO), and hydrogen gas (H_2_), are the major events [10–15]. Among these, NO is a multifunctional molecular gas, which can cross biological membranes [16]. In plants, it is considered as a regulator in response to various stresses, such as drought and osmotic stress [17, 18], salinity [11], heavy metal exposure [19], UV-B radiation [20], nanoparticles phytotoxicity [21, 22], and biotic stress [23, 24]. Biosynthesis of NO is catalyzed by nitrate reductase (NR), a well-known route of reductive reactions, and nitric oxide synthesis (NOS)-like biochemical pathway [25, 26]. Previously, there are more evidence showed that NO could modulate ROS generation, which is always accompanied by oxidative stress, to keep redox homeostasis and decrease oxidative damage [18, 27]. Specific plant proteins involved in metabolism, stress responses, and redox homeostasis, have also been identified as possible targets for *S*-nitrosylation, one of NO-dependent post-translational modifications [28, 29].

Methane (CH_4_) is not only the main element of nature gas and flammable ice, considered as a kind of clean fuel, but also have effects on anti-oxidative, anti-apoptotic and anti-inflammatory in animals [30–32]. It was further reported that formation of non-microbial CH_4_ in many different plant species is obviously increased by osmotic stress [33], high temperature [33, 34], UV-B radiation [33–35], physical injury [36]. Although the emission of CH_4_ from plant have been discovered for an extended period, the CH_4_ biosynthetic pathways related to CH_4_ formation and releasing, and its biological functions are still elusive [37]. Recent results showed that CH_4_ was able to induce cucumber adventitious rooting of hypocotyl cuttings (primary roots removed) [38, 39]. Similar to the response of NO, we proved that CH_4_ can alleviate salinity stress and copper stress in alfalfa plants [8, 40]. Recently, the alleviation of osmotic stress in maize seedlings by CH_4_ was confirmed, and the involvement of sugar and ascorbic acid metabolism was preliminarily elucidated [41]. However, the cross-talk between CH_4_ and NO signaling in plant tolerance against osmotic stress is still elusive.

To resolve above scientific question, in this study, time-course analysis of PEG-induced CH_4_ production was firstly determined. Considering that CH_4_ could form an explosive mixture with air, methane-rich water (MRW) was used to investigate the function of endogenous CH_4_ in plants [8, 38, 39]. Using this experimental approach, we provided pharmacological, physiological, and biochemical evidence to prove that CH_4_ could alleviate PEG-induced osmotic stress by modulating redox homeostasis and starch metabolism in mung bean. Importantly, this biological function was associated with the homeostasis of NO, a key cell signaling modulator [14–16]. The involvement of NO-triggered protein *S*-nitrosylation was also preliminarily suggested. Above results thus open a new window for CH_4_ signaling in plant kingdom.

## Methods

### Chemicals

All chemicals were purchased from Sigma (St Louis, MO, USA) unless otherwise stated. Polyethylene Glycol-6000 (PEG-6000) was purchased from Guangdong Guanghua Sci-Tech Co., Ltd, China, and is generally used to imitate osmotic stress. Sodium nitroprusside (SNP) was used as a well-known NO-releasing compound. The utterly light-inactivated SNP solution (old SNP) was used as a negative control. 2-(4-carboxyphenyl)-4,4,5,5-tetramethylimidazoline-1-oxyl-3-oxide potassium salt (cPTIO) was used as a scavenger of NO. Another scavenger of NO, 2-phenyl-4,4,5,5-tetramethylimidazoline-1-oxyl-3-oxide (PTIO) purchased from TCI company, was also used. Tungstate, a NR inhibitor, and *N*^ω^-nitro-_L_-Arg methyl ester hydrochloride (_L_-NAME), a mammalian NOS inhibitor, were used, respectively. In this study, the pilot experiments were carried out to determine the suitable concentrations of above chemicals with maximal responses.

### Preparation of methane-rich water (MRW) and determination of methane content

The CH_4_ gas (99.9%, v/v) from a compressed gas cylinder (Nanjing Special Gas Co., China) was bubbled into 500 ml distilled water with a rate of 160 ml min^-1^ for least 30 min at 25°C, thus reaching a saturated level. The corresponding methane-rich water was then immediately diluted with distilled water to different saturation required. The contents of CH_4_ in fresh methane-rich water (10, 50 and 100% saturation) were 0.13, 0.65, and 1.30 mM, respectively, and maintained at original concentration for at least 12 h.

For determining endogenous CH_4_ content, plant samples were treated according to the method described previously [41]. CH_4_ content was estimated using an Agilent 7820 model gas chromatograph (GC; Agilent Technologies Inc., USA) equipped with a flame ionization detector and a Porapak column (1/8 inch, 8 foot). The column was held isothermally at 70°C. The injection and detector temperature was adjusted to 200 °C and 300 °C, respectively. Nitrogen (N_2_) was used as the carrier gas, and air pressure was 0.5 MPa. The GC was calibrated using a standard CH_4_ mixture (2.0 ppm CH_4_ in N_2_; Nanjing Special Gas Co., China).

### Nitrogen and argon application

For nitrogen (N_2_) and argon (Ar) application, pure N_2_ and Ar gas from a gas cylinder (99.99%, Nanjing special gas Co., Ltd) were respectively bubbled into distilled water at the same rate as CH_4_ (160 ml min^-1^) for at least 30 min to obtain the same O_2_ concentration as methane-rich water (containing 1.3 mM CH_4_).

### Plant material and growth conditions

The healthy seeds of mung bean (*Vigna radiata* L. cv Jinlv No. 7) were selected and surface-sterilized with 5% NaClO for 10 min, followed by totally washed with distilled water and then dried. These seeds were presoaked with 20 ml of culture solution containing the indicated concentrations of CH_4_, N_2_, Ar, SNP, old SNP, PTIO, cPTIO, tungstate, and _L_-NAME, alone or their combinations. Then, these seeds were transferred to another Petri dishes and incubated on filter paper for 12 h at 25 °C in the darkness following the procedure described previously [42, 43]. The equal volume of 20% PEG-6000 was applied to mimic osmotic stress. All seeds were germinated in a growth chamber at 25°C with darkness. Treatment with distilled water was regarded as control (Con). After the indicated time points, the germinating seeds were rinsed with distilled water for three times. Then, the samples were harvested and used immediately, or frozen in liquid nitrogen and stored at -80°C for further analysis.

### Germination and growth analysis

Germination test was carried out with three independent experiments and at least three replicates for each. Each independent set of experiments was 120 seeds. Every Petri dish contains 40 seeds. After the indicated pretreatments followed by PEG-6000 stress for 48 h, germination rate (%) was recorded. Seeds were regarded as germinated when the emerging root was approximately the length of the seeds. Additionally, fresh weight (FW) and dry weight (DW) were detected after various treatments for 48 h.

### Detection of endogenous NO

According to previous report [15], about 200 μm transversal sections, which were obtained from root tips about 1 cm, were cut out at the indicated time points. These sections were incubated with 7.5 μM 4-amino-5-methylamino-2′,7′-difluorofluorescein diacetate (DAF-FM DA; a fairly specific NO fluorescent probe; prepared in 20 mM HEPES buffer, pH 7.8) for 10 min at 25°C in the darkness. Subsequently, the sections were washed three times with the same buffer for 15 min each, and monitored by laser scanning confocal microscopy (LSCM). The DAF-FM DA signal (excitation at 488 nm; emission at 500-530 nm) was captured as green fluorescence. All images were visualized using UltraVIEW VoX (PerkinElmer, Waltham, America). At least six individual samples were randomly selected and measured per treatment. The bright-field images corresponding to the fluorescent images were also shown. Fluorescence of NO production in root tissues (about an area of 150,000 μm^2^) was quantified based on 20 overlapping confocal planes of 2 μm each using the Volocity Demo software.

Besides, endogenous NO content was detected by Griess reagent assay [44] with some modifications. About 2000 mg samples were homogenized in a mortar with 50 mM cool acetic acid buffer, and then centrifuged. After various treatments, samples were pre-incubated in 100 μM cPTIO, a specific scavenger of NO, for 1 h, were regarded as the blank control. The supernatant was collected and reacted with Griess reagent for 30 min. Absorbance was assayed at 540 nm, and the NO content was calculated by in comparison with a standard curve of NaNO_2_.

### Endogenous ROS detection by LSCM

For determining endogenous ROS, about 200 μm transversal sections from the root tips were incubated with 10 μM 2’,7’-dichlorofluorescein diacetate (H_2_DCF-DA, a fairly specific ROS fluorescent probe) [45] in 20 mM HEPES buffer (pH 7.8) for 10 min. The H_2_DCF-DA signal (excitation at 488 nm; emission at 500-530 nm) was captured as green fluorescence, and monitored by LSCM.

### Histochemical staining and determination of thiobarbituric acid reactive substances (TBARS)

After various treatments, whole roots were rinsed with distilled water for three times, and then dried with filter papers and immediately soaked in the specific reagents. Histochemical detection of lipid peroxidation was performed with Schiff's reagent [46]. All stained roots were observed under a light microscope (model Stemi 2000-C; Carl Zeiss, Germany), and photographed (Powershot A620, Canon Photo Film, Japan).

Lipid peroxidation was detected by measuring the concentration of thiobarbituric acid reactive substances (TBARS) as described previously [46]. Briefly, about 1000 mg of germinating seeds was homogenized in a mortar with 10 ml solution containing 0.25% 2-thiobarbituric acid (TBA) and 10% trichloroacetic acid (TCA). After heating at 95 °C for 30 min, the mixture was quickly cooled in an ice bath, and centrifuged at 10,000 × g for 10 min. The absorbance of the supernatant was read at 532 nm and corrected for unspecific turbidity by subtracting the absorbance at 600 nm. The blank was 0.25% TBA in 10% TCA. The results were expressed as nmol g^-1^ fresh weight (FW).

### Determination of reducing sugar, soluble sugar content, *α*-amylase and amylase activities

For detected reducing and soluble sugar contents, α-amylase and total amylase activities, the germinating seeds were homogenized in a mortar. Reducing sugar and soluble sugar contents were estimated following the previous methods [47]. *α*-amylase and total amylase activities were detected according to the methods described previously [48].

### Extraction of total protein

For protein extraction, germinating seeds were homogenized in a mortar with liquid nitrogen to fine powder, and then re-suspended in HEN buffer containing 250 mM Hepes-NaOH (pH 7.7), 1 mM EDTA, and 0.1 mM protease inhibitor cocktail, and centrifuged at 13,000 g for 20 min at 4°C. The supernatants were transferred to clean tube stored at 4°C for Western blotting analysis.

### Western blotting analyses of protein *S*-nitrosylation

According to the previous protocols [39], analysis of protein *S*-nitrosylation was carried out. The biotin-labeled protein samples were separated under non-reducing conditions by 12% SDS-PAGE for 1.5 h at 120 V. After blotting onto a polyvinylidene difluoride (PVDF) membrane, anti-biotin antibody (HRP; Abcam antibodies, Cambridge, UK) was added at 1:10,000 dilution for 1 h at room temperature. Meanwhile, as a loading control, parallel sets of gels were stained with colloidal Coomassie blue to confirm that the loaded proteins were equal amounts.

### Statistical analyses

All date expressed are the mean values ± SE of three independent experiments with at least three replicates for each. Statistical analysis was performed using SPSS 16.0 software. For statistical analysis, one-way analysis of variance (ANOVA) followed by Duncan's multiple range test (*P* < 0.05) was chosen.

## Results

### Osmotic stress induces CH_4_ production in a time-dependent fashion

**Fig. 1:**
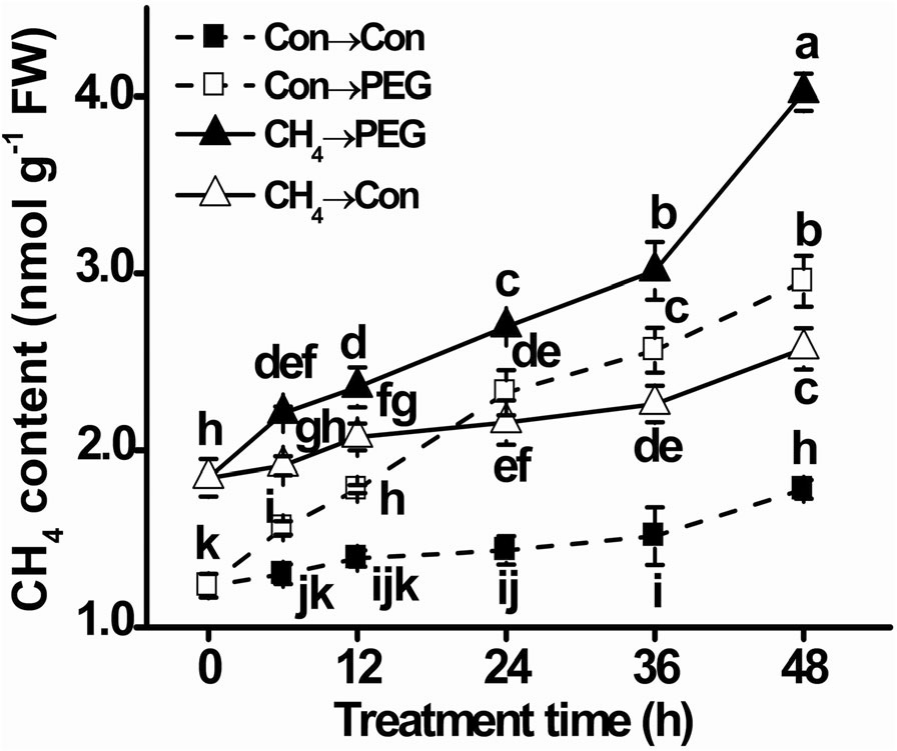
Time-dependent changes of CH_4_ production. Mung bean seeds presoaked with solution containing 1.3 mM CH_4_ for 12 h, were then shifted to 20% PEG-6000 for another 48 h. The endogenous CH_4_ production in germinating seeds was then detected by gas chromatography (GC). Treatment with distilled water was regarded as control (Con). Data are means ± SE of three independent experiments with three replicates for each. Bars with different letters denote significant difference at *P* < 0.05 according to Duncan’s multiple range test

In order to evaluate whether osmotic stress could induce the production of CH_4_, CH_4_ content was analyzed in germinating mung bean seeds by gas chromatography (GC). Fig. 1 showed that, in comparison with control samples, PEG stress resulted in a gradual and significant increase in CH_4_ production during a 48 h period of treatment, suggesting the possible role of endogenous CH_4_ in osmotic stress responses.

### PEG-induced inhibition of seed germination was alleviated by CH_4_ and sodium nitroprusside (SNP)

**Fig. 2:**
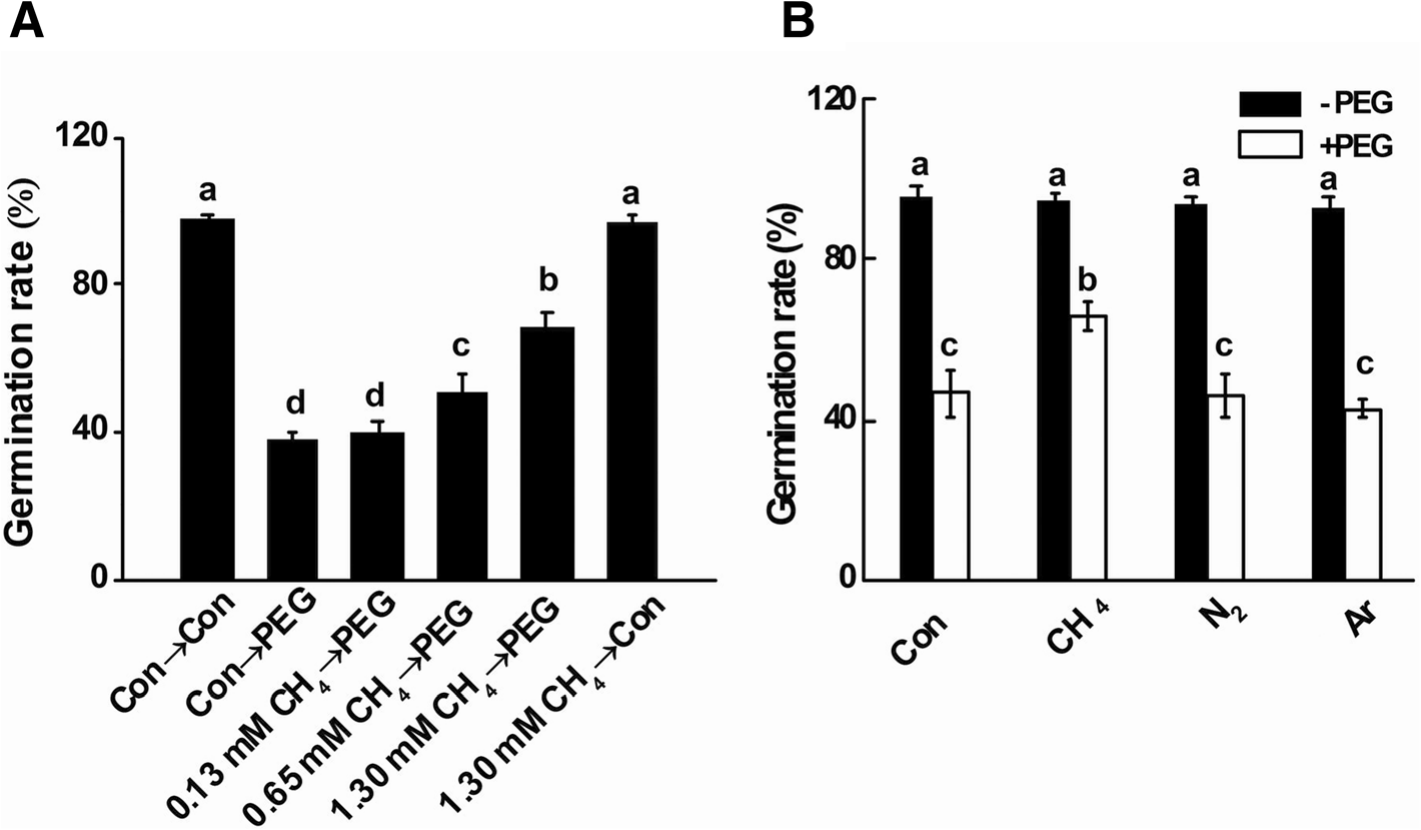
Unlike the responses of CH_4_ (**a**), hypoxia (**b**) failed to alleviate inhibition of seed germination caused by PEG. Mung bean seeds presoaked with solutions containing the indicated concentrations of CH_4_, nitrogen (N_2_), or argon (Ar) for 12 h, were then shifted to 20% PEG-6000 for another 48 h. Afterwards, germination rates were determined. Treatment with distilled water was regarded as control (Con). Data are means ± SE of three independent experiments with at least three replicates for each. Bars with different letters denote significant difference at *P* < 0.05 according to Duncan’s multiple range test

To characterize the effect of CH_4_ on osmotic stress, culture medium containing different concentrations of CH_4_ (ranging from 0.13 to 1.30 mM) were applied. As showed in Fig. 2a, the inhibition of seed germination was observed in PEG-treated sample. Further results illustrated that CH_4_ pretreatment was effective in reversing the negative impact of PEG stress on seed germination in a dose-dependent manner, with 1.30 mM in particular.

Results shown in Fig. 1 further revealed that 1.3 mM CH_4_ pretreatment for 12 h (0 h; the beginning of osmotic stress) brought about a 49.2% increase in CH_4_ production. Subsequent stress (CH_4_→PEG) aggravated CH_4_ production, compared to stress alone. Based on the above findings, 1.3 mM CH_4_ was used in the subsequent experiment.

To rule out the possibility that CH_4_-promoted role might be partly due to hypoxia, culture medium containing nitrogen gas (N_2_) and inert gas argon (Ar) was subsequently applied. As expected, unlike the beneficial response of CH_4_, both N_2_ and Ar failed to alleviate PEG-triggered seed germination inhibition (Fig. 2b).

**Fig. 3:**
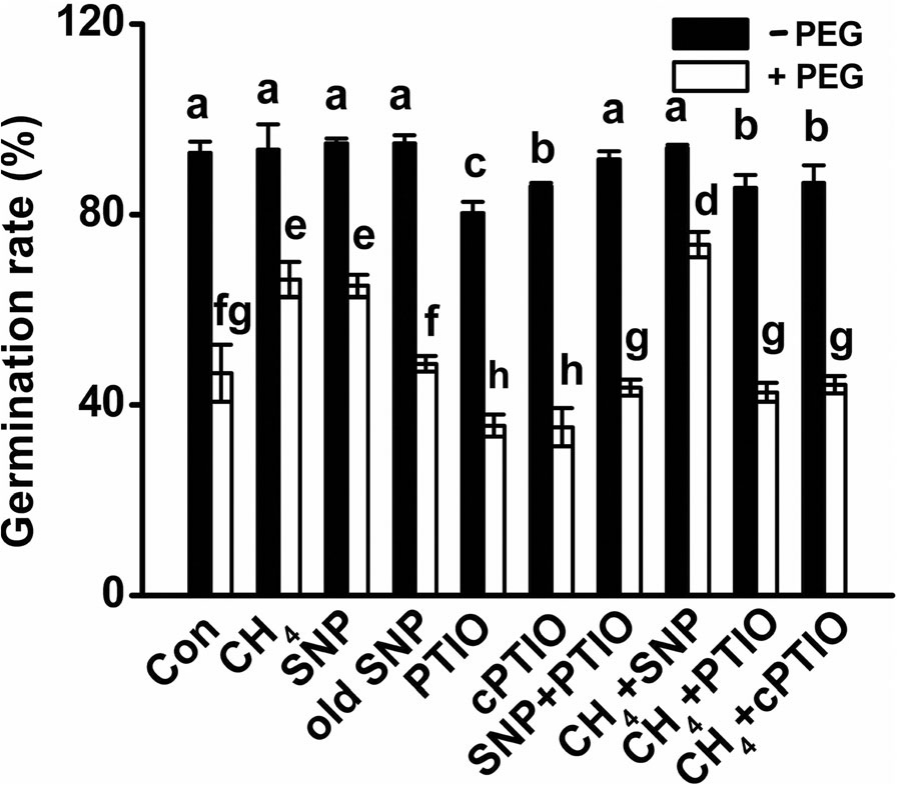
CH_4_-induced alleviation of seed germination inhibition caused by PEG stress was sensitive to PTIO and cPTIO. Mung bean seeds presoaked with solutions containing 1.3 mM CH_4_, 100 μM SNP, 100 μM old SNP, 500 μM PTIO, 500 μM cPTIO, alone or their combinations for 12 h, were then shifted to 20% PEG-6000 for another 48 h. Afterwards, germination rates were determined. Treatment with distilled water was regarded as control (Con). Data are means ± SE of three independent experiments with at least three replicates for each. Bars with different letters denote significant difference at *P* < 0.05 according to Duncan’s multiple range test

It was also noticed that the application of sodium nitroprusside (SNP; a NO-releasing compound) (Fig. 3), brought about the significant alleviation in the seed germination inhibition caused by PEG stress. Above response was not observed in the pretreatment with old SNP solution (a negative control of SNP, containing no NO, but ferrocyanide, nitrate and nitrite), suggesting that the beneficial role of SNP was NO-dependent.

### CH_4_-alleviated seed germination inhibition was sensitive to cPTIO and PTIO, two scavengers of NO

To investigate the possible involvement of NO in above CH_4_ response, two specific scavengers of NO, 2-phenyl-4,4,5,5-tetramethylimidazoline-1-oxyl-3-oxide (PTIO) and 2-(4-carboxyphenyl)-4,4,5,5-tetramethylimidazoline-1-oxyl-3-oxide potassium salt (cPTIO), were used. Interestingly, we observed that CH_4_-induced alleviation of seed germination inhibition was significantly blocked by the addition of cPTIO and PTIO, respectively (Fig. 3). The cotreatment with PTIO suppressed the inducible effect of SNP on the alleviation of seed germination inhibition as well. The additive behavior appeared when CH_4_ was added together with SNP followed by stress. These results, together with the performances of old SNP (Fig. 3), suggested the importance role of NO in the beneficial responses triggered by CH_4_. In view of the relative expensive price of cPTIO and higher amount of scavenger required for a fully assay, we adopted PTIO as a NO scavenger in the subsequent investigation.

### NO might be involved in CH_4_-modulated starch metabolism in response to osmotic stress

To investigate the mechanism of CH_4_-alleviated seed germination inhibition, several physiological parameters, including reducing and soluble sugar contents, *α*-amylase and total amylase activities, were detected. As shown in Table 1, PEG stress for 48 h resulted in obvious decline of those parameters in germinating seeds. The combination of PEG with CH_4_ or SNP pretreatment enhanced *α*-amylase and total amylase activities, both of which were in accordance with the increased contents of reducing sugar and soluble sugar. On the contrary, above changes in starch metabolism triggered by CH_4_ and SNP were impaired by the addition of PTIO. Alone, PTIO decreased above parameters, suggesting the possible role of endogenous NO.

**Table 1:** Both CH_4_- and NO-alleviated sugar metabolism inhibition caused by PEG stress were sensitive to PTIO

Treatments	Reducing sugar content (mg g^-1^ DW)	Soluble sugar content (mg g^-1^ DW)	*α*-amylase activity (mg min^-1^g^-1^ DW)	Amylase activity (mg min^-1^g^-1^ DW)
Con→Con	31.76 ± 1.80^b^	18.60 ± 0.37^b^	68.36 ± 5.16^b^	429.37 ± 14.11^b^
Con→PEG	20.71 ± 0.77^f^	14.25 ± 0.77^f^	21.28 ± 2.18^f^	173.68 ± 4.19^f^
CH_4_→PEG	28.22 ± 0.45^d^	16.77 ± 0.32^d^	35.16 ± 3.25^d^	275.48 ± 7.84^d^
CH_4_→Con	34.02 ± 0.78^a^	21.11 ± 0.56^a^	79.40 ± 5.05^a^	474.73 ± 16.27^a^
CH_4_+PTIO→PEG	26.65 ± 0.47^e^	15.85 ± 0.33^e^	25.06 ± 2.83^e^	219.84 ± 2.07^e^
CH_4_+PTIO→Con	30.87 ± 0.51^b^	18.29 ± 0.28^b^	63.14 ± 4.99^bc^	412.25 ± 47.67^bc^
SNP→PEG	28.67 ± 0.86^d^	16.50 ± 0.82^d^	38.82 ± 1.85^d^	280.53 ± 10.74^d^
SNP→Con	34.32 ± 1.88^a^	21.32 ± 1.34^a^	84.54 ± 3.93^a^	488.89 ± 11.33^a^
SNP+PTIO→PEG	26.18 ± 1.19^e^	15.52 ± 0.05^e^	26.30 ± 4.20^e^	233.44 ± 7.07^e^
SNP+PTIO→Con	30.37 ± 1.01^b^	18.37 ± 0.93^b^	62.00 ± 4.81^bc^	412.83 ± 46.29^bc^
PTIO→PEG	18.86 ± 0.99^g^	12.74 ± 0.55^g^	12.87 ± 3.01^g^	127.62 ± 9.15^g^
PTIO→Con	29.94 ± 0.59^c^	17.28 ± 0.52^c^	60.43 ± 0.50^c^	393.47 ± 10.31^c^

Mung bean seeds were presoaked with solutions containing 1.3 mM CH_4_, 100 μM SNP, 500 μM PTIO, alone or their combinations for 12 h, and then shifted to 20% PEG-6000 for another 48 h. Afterwards, reducing and soluble sugar contents, *α*-amylase and total amylase activities in germinating seeds, were determined. Treatment with distilled water was regarded as control (Con). Within each set of experiments, data are means ± SE of three independent experiments with at least three replicates for each. Bars with different letters denote significant difference at *P* < 0.05 according to Duncan’s multiple test

### CH_4_-mediated endogenous NO generation was reversed by tungstate and _L_-NAME, two synthetic inhibitors of NO

**Fig. 4:**
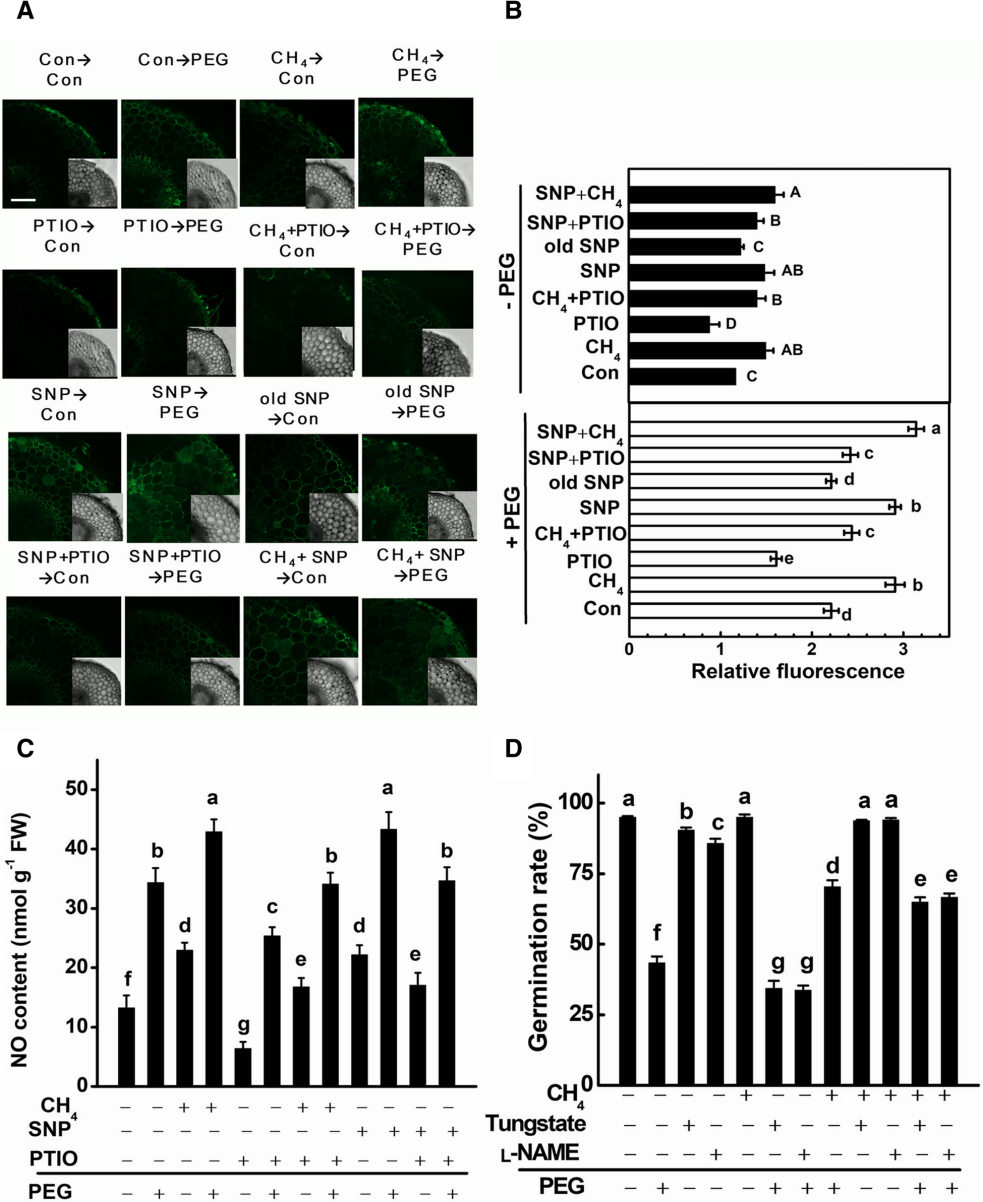
CH_4_- and PEG-induced NO production was sensitive to the PTIO, and the alleviation caused by CH_4_ was sensitive to tungstate and _L_-NAME. Mung bean seeds were presoaked with solutions containing 1.3 mM CH_4_, 100 μM SNP, 100 μM old SNP, 500 μM PTIO, 1 mM tungstate, 500 μM _L_-NAME, alone or their combinations for 12 h, and then shifted to 20% PEG-6000 for another 12 h. Afterwards, about 200 μm transversal sections from the root tips were loaded with 4-amino-5-methylamino-2’,7’-difluorofluorescein diacetate (DAF-FM DA), and detected by laser scanning confocal microscopy (LSCM; **a**). Bars = 25 μm. DAF-FM DA fluorescence densities according to (**a**) were also given (**b**). At least six individual samples were randomly selected and measured per treatment. Meanwhile, the content of NO in root tissues was determined by Griess reagent assay (**c**). Besides, the relationship between CH_4_ and tungstate, _L_-NAME was also analysed (**d**). Treatment with distilled water was regarded as control (Con). Data are means ± SE of three independent experiments with at least three replicates for each. Bars with different letters denote significant difference at *P* < 0.05 according to Duncan’s multiple range test

In order to assess the role of NO in the physiological role of CH_4_, endogenous NO levels in root tissues were checked by using the permeable NO-sensitive fluorophore 4-amino-5-methylamino-2’7’-difluorofluorescein diacetate (DAF-FM DA) in combination with laser scanning confocal microscopy (LSCM). We compared fluorescence detected in the presence of SNP, old SNP, and PTIO. As expected, in the presence (in particularly) or absence of PEG stress conditions, SNP-induced fluorescence was differentially reduced when root tissues were co-pretreated with PTIO (Fig. 4a, b). Unlike SNP, old SNP failed to influence PEG-induced DAF-FM DA green fluorescence.

Under osmotic stress conditions, the CH_4_-triggered induction of DAF-FM DA fluorescence was markedly abolished by the removal of NO with PTIO, suggesting that above strengthened-fluorescence was NO-specific (Fig. 4a, b). The obtained data with Griess reagent assay (Fig. 4c) were in line with those of DAF-FM-associated fluorescence, further confirming that the DAF-FM-dependent fluorescence was related to NO levels in vivo. Combined with corresponding phenotypes (Fig. 3), these result apparently supported the idea that NO production might be involved in CH_4_-induced tolerance against PEG stress.

To better characterize the main source(s) of NO generated by CH_4_ in stressed mung bean, seeds were pretreated with chemicals that interfere with NO production before osmotic stress. In our experiment, tungstate and _L_-NAME were used. Tungstate is the inhibitor of NR [15], and _L_-NAME is the inhibitor of mammalian NOS, which was usually applied to inhibit plant NOS-like activity [39, 49]. As shown in Fig. 4d, tungstate significantly blocked CH_4_-alleviated seed germination inhibition, suggesting that CH_4_-triggered NO production might be partly resulted from NR. Comparatively, _L_-NAME had a lesser, yet significant influence on the CH_4_-triggerd response, implying that NOS-like enzyme might be partly involved.

### CH_4_-modualted redox homeostasis was sensitive to the removal of endogenous NO

**Fig. 5:**
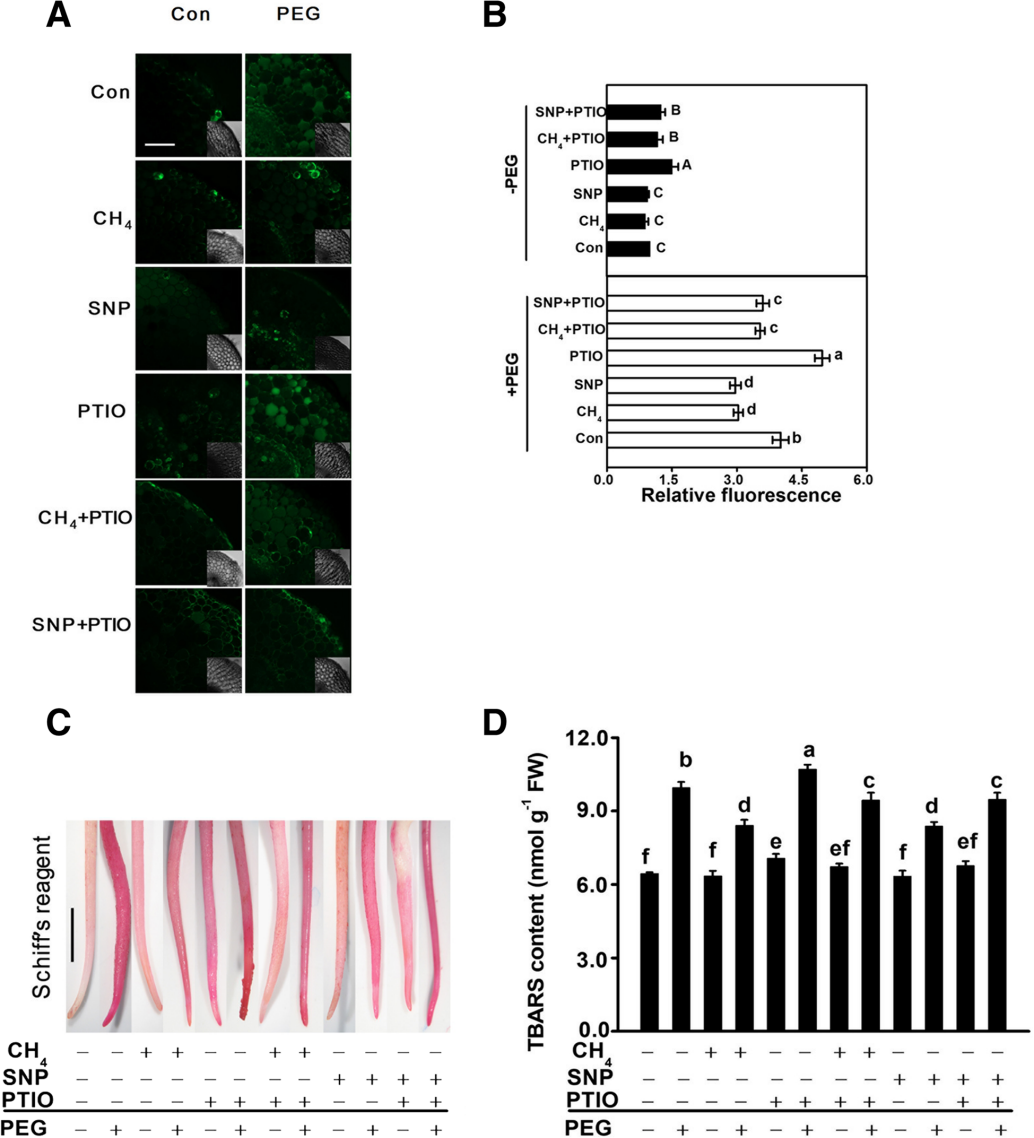
CH_4_-reestablished redox homeostasis was impaired by the removal of NO. Mung bean seeds were presoaked with solutions containing 1.3 mM CH_4_, 100 μM SNP, 500 μM PTIO, alone or their combinations for 12 h, and then shifted to 20% PEG-6000 for another 12 h. Afterwards, about 200 μm transversal sections from the root tips were loaded with H_2_DCF-DA, and detected by laser scanning confocal microscopy (LSCM; **a**). Bars = 25 μm. H_2_DCF-DA fluorescence densities according to (**a**) were also given (**b**). Meanwhile, the roots were stained with Schiff's reagent (**c**), and immediately photographed under a light microscope. Bars: 1 mm. Meanwhile, the content of TBARS was determined (**d**). At least six individual samples were randomly selected and measured per treatment. Treatment with distilled water was regarded as control (Con). Bars with different letters denote significant difference at *P* < 0.05 according to Duncan’s multiple range test

Upon stress conditions, redox homeostasis is impaired, and NO-mediated plant tolerance against stress is normally associated with the reestablishment of redox homeostasis [50]. To further investigate the mechanism of CH_4_-elicited tolerance against osmotic stress, we analyzed whether redox homeostasis was involved, in a manner similar to NO response. Sections detached from root tips were stained with H_2_DCF-DA (a probe for ROS), and LSCM was used to check changes in intracellular ROS levels (Fig. 5a, b). As expected, ROS overproduction occurred when PEG was supplemented, confirming that redox imbalance happened. PTIO alone induced redox imbalance as well. Further results revealed that PEG-triggered redox imbalance was markedly blocked by the addition of CH_4_, which was abolished by PTIO. Similar responses were observed when SNP was applied. Above results suggested that CH_4_-modualted redox homeostasis was sensitive to the removal of endogenous NO.

To confirm above deduction, a histochemical staining by Schiff’s reagent, which is used to monitor the level of peroxidation of membrane lipids, was performed (Fig. 5c). Compared with the control samples, the roots of mung bean treated with PEG alone or pretreated with PTIO alone were stained extensively. Those pretreated with CH_4_ or SNP followed by stress showed a less staining, which were markedly reversed when PTIO was cotreated together. Meanwhile, changes in TBARS contents exhibited the similar tendencies (Fig. 5d).

### The possible involvement of CH_4_-triggered NO-mediated *S*-nitrosylation

**Fig. 6:**
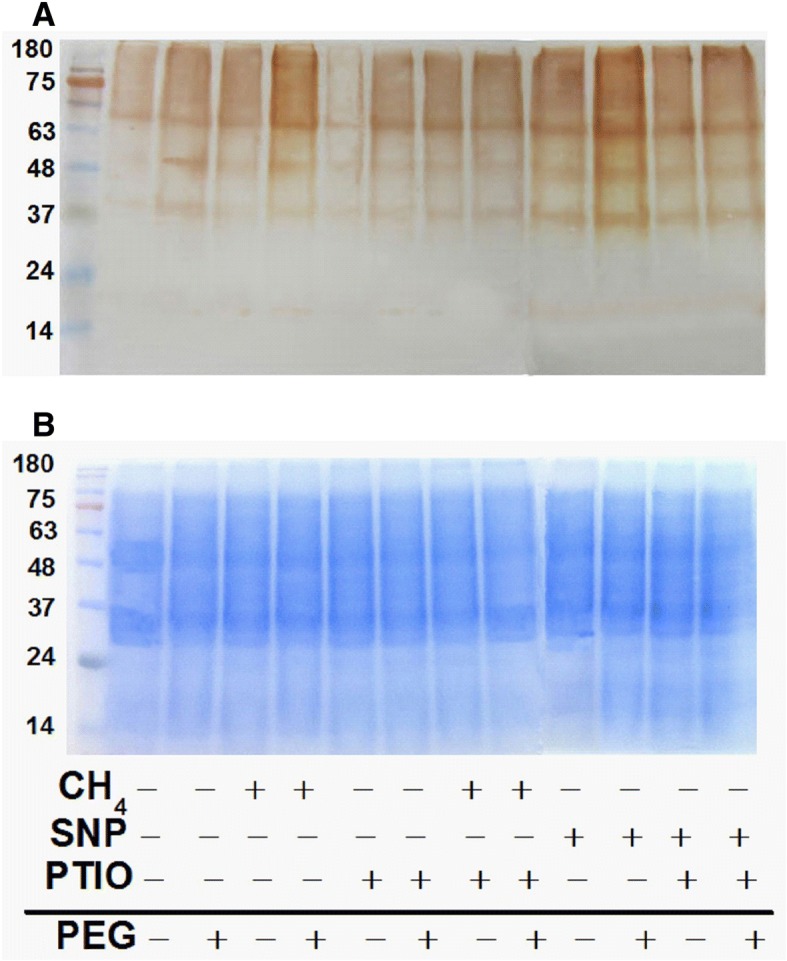
In vivo analysis revealed that CH_4_-mediated *S*-nitrosylation was sensitive to PTIO. Mung bean seeds were presoaked with solutions containing 1.3 mM CH_4_, 100 μM SNP, 500 μM PTIO, alone or their combinations for 12 h, and then shifted to 20% PEG-6000 for another 48 h. Biotin labeled proteins were detected by Western blot with anti-biotin antibodies (**a**). Meanwhile, the equal protein amounts were evaluated by Coomassie staining (**b**) after SDS-PAGE separation. Numbers on the left of the panels indicate the position of the protein markers in kDa. The gels are representative of at lest five replicates per experiment

To further understand the possible role of CH_4_ in protein level, the NO-mediated *S*-nitrosylated protein level was detected by using the protein extracted from mung bean and the modified biotin switch assay. Fig. 6 showed that similar to the responses of SNP alone, stress stimulated nitrosylation, which was strengthened by CH_4_ or SNP. By contrast, above CH_4_- or SNP-stimulated nitrosylation in stressed plants were obviously abolished when endogenous NO was removal with PTIO. Alone, the pretreatment with PTIO could decrease nitrosylation levels in the presence or absence of PEG.

## Discussion

Here, we provided the molecular basis of CH_4_-induced plant tolerance against osmotic stress: the involvement of NO signaling.

Similar to our previous findings in maize root tissues upon osmotic stress [41], this report revealed that an increase in the concentration of CH_4_ is one of the earliest responses involved in the signaling cascade triggered by PEG stress in germinating mung bean seeds (Fig. 1). Although the biochemical routes responsible for plant CH_4_ production have not elucidated in this report, our finding that PEG triggered CH_4_ production was in agreement with those obtained in germinating alfalfa seeds subjected to copper stress [40] and salinity [8]. Combined with the results showing that osmotic stress obviously increased CH_4_ emission in pea leaves [33], and CH_4_ might be associated with adventitious rooting in cucumber [38, 39], we therefore deduced that CH_4_ might be produced and then emitted by plants as a consequence of osmotic stress, and this might be a universal event, or stress- and even developmental stage-specific in different plant species. The possible role(s) of endogenous CH_4_ production was subsequently investigated in PEG-stressed mung bean.

The physiological function of CH_4_ was firstly recognized in animals [31], showing that it displays the protective response against the intestinal ischemic/reperfusion (IR) injury-induced oxidative stress and inflammation. Until now, it was recognized that CH_4_ serves as multiple functions in animals via anti-oxidative, anti-apoptotic and anti-inflammatory actions [30, 32, 51]. Similar to the beneficial roles of NO against osmotic stress and drought in plants [17, 18], by using culture solutions containing different concentrations of CH_4_, we provided evidence, showing that CH_4_ could participate in the promotion of plant tolerance against osmotic stress in mung bean during germination.

First, PEG-induced inhibition of seed germination was alleviated by CH_4_ in a dose-dependent fashion, with 1.3 mM in maximum response (Fig. 2a). Above beneficial role could be explained by CH_4_-induced *α*-amylase and total amylase activities, thus accelerating the formation of reducing and total sugar (Table 1). These results were consistent with our recent report [41], showing that PEG-induced CH_4_ production was more pronounced in the drought stress-tolerant maize cultivar than stress-sensitive cultivar. Our subsequent experiments confirmed that the main factor of CH_4_-rich solution responsible for the alleviation of seed germination inhibition was the dissolved CH_4_, rather than hypoxia (Fig. 2b). Similar results were confirmed in hydrogen-rich water-mediated tolerance against drought in Arabidopsis [45]. In view of the fact that the inhibition of seed germination is one of the representative phenotypes in response to osmotic stress, we therefore confirmed that CH_4_ enhances plant tolerance against osmotic stress. Similar rescuing responses in salinity [8] and heavy metal stress [40] were previously reported, although the specific mechanisms are still not fully elucidated.

It has been well documented that gaseous signaling molecules have not only discrete, but also overlapping roles in conferring plant stress tolerance [11, 17, 25]. In the subsequent work, we confirmed that the beneficial roles of CH_4_ are, at least partly, dependent on the action of NO, a well-known gaseous signaling molecule in plants [16, 23]. First, CH_4_ strengthened the increase in NO production in roots upon PEG stress (Fig. 4a–c). Above mentioned CH_4_-triggered NO production was markedly impaired by the addition of PTIO (a scavenger of NO), which was confirmed by the combination of LSCM and Griess reagent assay. Similar phenomenon occurred when SNP was applied in the presence of PEG and PTIO. Meanwhile, related phenotypes in terms of the alleviation of seed germination inhibition (Fig. 3) and corresponding parameters (Table 1) were reversed. Third, unlike the responses of SNP, old SNP failed to influence above parameters. These results, together with our previous results [39], suggested the novel function of NO in the beneficial roles of CH_4_ in stressed conditions and different developmental processes. Additionally, the possible involvement of NR and NOS-like protein in CH_4_-induced NO production was preliminarily corroborated by the findings that corresponding inhibitors (tungstate and _L_-NAME) inhibited CH_4_-alleviated seed germination inhibition caused by PEG (Fig. 4d). In fact, the contribution of NR is very difficult to evaluate in our experimental conditions, since tungstate, an inhibitor of NR, is also a ABA synthetic inhibitor. Thus, further genetic evidence should be considered. Certainly, other route(s) responsible for NO production might be another consideration.

Keeping redox homeostasis is an important mechanism for plant tolerance against osmotic stress [7]. Upon PEG stress, redox imbalance occurred. For instances, PEG stress could obviously induce ROS overproduction (Fig. 5a, b) and oxidative damage (Fig. 5c, d). These responses could be alleviated by CH_4_. It was further observed that CH_4_-reestablished redox balance was NO-dependent, since PTIO could counteract the effect of CH_4_. Together, above results indicated that CH_4_-evoked endogenous NO production in mung bean was positively correlated with plant tolerance against osmotic stress, and the reestablishment of redox homeostasis was an important mechanism.

It was well-known that NO-based *S*-nitrosylation is a highly conserved protein posttranslational modification that regulates diverse biological processes [39]. To further confirm the role of NO in the actions of CH_4_, the combination with pharmacological approach and biotin switch method was used (Fig. 6). Consistent with the previous results in cucumber explants [39], our results suggested the role of NO-mediated *S*-nitrosylation in CH_4_ responses, which might be in a stress- and development-specific fashion [28].

## Conclusion

**Fig. 7:**
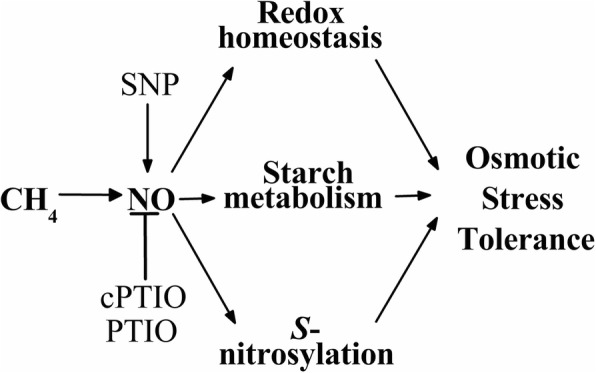
Schematic representation of the signaling pathway involving CH_4_, NO, reestablishment of redox homeostasis, starch metabolism and *S*-nitrosylation, during osmotic stress tolerance. The signaling cascade showed that the beneficial role of CH_4_ was in a NO-dependent fashion. T bar, inhibition

Taken together, our results define a main branch of NO-regulated redox homeostasis and starch metabolism involved in the CH_4_ signaling cascade during plant tolerance against osmotic stress (Fig. 7). CH_4_-governed NO-mediated *S*-nitrosylation might be an interesting mechanism. Therefore, the identification of NO-targeted nitrosylated protein(s) by using nanoLC/MS/MS might help us to understand the detailed mechanism of CH_4_ action.

## Abbreviations

Ar: Argon
CH_4_: methane
CO: Carbon monoxide
cPTIO: 2-(4-carboxyphenyl)-4,4,5,5-tetramethylimidazoline-1-oxyl-3-oxide potassium salt
DAF-FM DA: 4-amino-5-methylamino-2′,7′-difluorofluorescein diacetate
DW: Dry weight
FW: Fresh weight
GC: Gas chromatograph
H_2_: hydrogen gas
H_2_DCF-DA: 2’,7’-dichlorofluorescein diacetate
H_2_S: hydrogen sulfide
_L_-NAME: *N*^ω^-nitro-_L_-Arg methyl ester hydrochloride
LSCM: Laser scanning confocal microscopy
MRW: Methane-rich water
N_2_: Nitrogen
NO: Nitric oxide
NOS: Nitric oxide synthesis
NR: Nitrate reductase
PEG: Polyethylene glycol
PTIO: 2-phenyl-4,4,5,5-tetramethylimidazoline-1-oxyl-3-oxide
PVDF: Polyvinylidene difluoride
ROS: Reactive oxygen species
SNP: Sodium nitroprusside
TBA: 2-thiobarbituric acid
TBARS: Thiobarbituric acid reactive substances
TCA: Trichloroacetic acid
